# Intradermal Indocyanine Green for *In Vivo* Fluorescence Laser Scanning Microscopy of Human Skin: A Pilot Study

**DOI:** 10.1371/journal.pone.0023972

**Published:** 2011-08-31

**Authors:** Constanze Jonak, Hans Skvara, Rainer Kunstfeld, Franz Trautinger, Johannes A. Schmid

**Affiliations:** 1 Division of General Dermatology, Department of Dermatology, Medical University of Vienna, Vienna, Austria; 2 Karl Landsteiner Institute for Dermatological Research, Landesklinikum St. Pölten, St. Pölten, Austria; 3 Division of Immunology, Allergy and Infectious Diseases, Department of Dermatology, Medical University of Vienna, Vienna, Austria; 4 Department of Vascular Biology and Thrombosis Research, Center for Physiology and Pharmacology, Medical University Vienna, Vienna, Austria; The University of Queensland, Australia

## Abstract

**Background:**

In clinical diagnostics, as well as in routine dermatology, the increased need for non-invasive diagnosis is currently satisfied by reflectance laser scanning microscopy. However, this technique has some limitations as it relies solely on differences in the reflection properties of epidermal and dermal structures. To date, the superior method of fluorescence laser scanning microscopy is not generally applied in dermatology and predominantly restricted to fluorescein as fluorescent tracer, which has a number of limitations. Therefore, we searched for an alternative fluorophore matching a novel skin imaging device to advance this promising diagnostic approach.

**Methodology/Principal Findings:**

Using a Vivascope®-1500 Multilaser microscope, we found that the fluorophore Indocyanine-Green (ICG) is well suited as a fluorescent marker for skin imaging *in vivo* after intradermal injection. ICG is one of few fluorescent dyes approved for use in humans. Its fluorescence properties are compatible with the application of a near-infrared laser, which penetrates deeper into the tissue than the standard 488 nm laser for fluorescein. ICG-fluorescence turned out to be much more stable than fluorescein *in vivo*, persisting for more than 48 hours without significant photobleaching whereas fluorescein fades within 2 hours. The well-defined intercellular staining pattern of ICG allows automated cell-recognition algorithms, which we accomplished with the free software *CellProfiler*, providing the possibility of quantitative high-content imaging. Furthermore, we demonstrate the superiority of ICG-based fluorescence microscopy for selected skin pathologies, including dermal nevi, irritant contact dermatitis and necrotic skin.

**Conclusions/Significance:**

Our results introduce a novel *in vivo* skin imaging technique using ICG, which delivers a stable intercellular fluorescence signal ideal for morphological assessment down to sub-cellular detail. The application of ICG in combination with the near infrared laser opens new ways for minimal-invasive diagnosis and monitoring of skin disorders.

## Introduction

*In vivo* confocal laser scanning microscopy is used in well-equipped dermatology departments as a state-of-the-art investigative tool in skin research as well as in clinical routine [1], [2], [3]. This imaging technique allows for non-invasive monitoring of the skin with microscopic resolution second only to histological analysis. Physiological and pathological skin conditions of the same body site can be recorded, as well as monitored along with treatment [4], [5]. The method employs the reflected light after laser illumination of the tissue to obtain morphological information. This is based on the fact that light is reflected differentially according to the specific refractive indices (e.g. melanin, keratin, lipid, water, or collagen) within the skin, producing the required contrast [6], [7]. Similar to computerized tomography, reflectance is detected only from the focal plane via a pinhole. In this confocal manner images of thin optical sections of horizontal tissue are gained *in vivo*, revealing cellular and even sub-cellular detail [8], thereby lending this technique the term reflectance confocal microscopy (RCM). Since the inception of this technique in 1995 [7] RCM has been amply used in dermatology for the diagnosis of skin tumors as well as inflammatory skin disorders [9], [10], [11], [12], [13]. It is already used in the routine setting in some specialized centers.

Incorporation of the principle of fluorescence to this skin imaging technique led to fluorescence confocal microscopy (FCM). This technique depends on exogenously added fluorescent molecules (fluorophores) applied into the tissue and on an appropriate laser light for the excitation to generate the image contrast. Only a few reports on FCM in humans exist, and this is compounded by the fact that just a very limited number of commercially available fluorescent dyes are approved for application in humans [14], [15]. The majority of published data on FCM *in vivo* was obtained using a device termed Stratum® (Optiscan Ltd, Melbourne, Australia), which is based on a 488 nm argon laser [16]. To our knowledge, the only dye used, with this equipment thus far was fluorescein, which is approved by the U.S. Food and Drug Administration (FDA) for *in vivo* use [17], [18]. A major limitation of this method is that illumination of tissue at 488 nm is restricted to a small depth within the skin (approximately reaching the top of the papillary dermis) because this wavelength is strongly absorbed by biological tissue. Another drawback is that the detectable fluorescence signal of fluorescein decays quickly, with a peak reached after a few minutes and a near-complete loss of the signal following 25–40 min [15].

This limitation is bypassed with newer systems that include additional laser lines, such as the Vivascope® 1500 Multilaser, which incorporates three different lasers with wavelengths of 488 nm (blue), 658 nm (red) and 785 nm (near-infrared) that can be used both in reflectance- as well as in fluorescence mode. As intimated above, the Multilaser compensates for the fact that shorter wavelengths are limited in penetration depth thereby providing higher resolution only for upper cellular layers, whereas longer wavelengths penetrate deeper into the skin due to lower absorbance by biological material. Near infrared illumination allows for examination at a maximum depth reaching the upper reticular dermis [19].

Here, we report on the use of Indocyanine green (ICG) fluorescence for generating images of human skin *in vivo* after its intradermal application. ICG is a water-soluble tricarbocyanine dye. Since 1959 its intravenous application is FDA-approved for diagnostic purpose (e.g. determination of cardiac output, liver function diagnostics and ophthalmic angiography) [20], [21]. Recently published data show that ICG injection is a safe and highly sensitive method for sentinel lymph node detection in breast cancer patients [22]. In our study, we compared the *in vivo* kinetics of ICG with that of fluorescein and moreover we tested the extent of ICG photobleaching. Finally we assessed this method for its application in the diagnosis of skin pathologies such as dermal nevus, irritant contact dermatitis or necrosis.

Our data indicate that ICG is far superior to fluorescein in fluorescence confocal microscopy of skin *in vivo* and suggest using this dye in this promising new diagnostic imaging technique.

## Materials and Methods

### Participants

9 healthy volunteers, 5 women and 4 men, aged between 20 and 57 years (mean 36 years) were recruited for testing the applicability of ICG. Five of them were additionally appointed as fluorescein controls. Furthermore, 3 patients respectively suffering from irritant contact dermatitis, necrosis due to KTP-laser treatment and a dermal nevus were investigated. All individuals were Caucasian, with skin types ranging from II to IV. Healthy skin without clinical alteration on the volar forearm (n = 9) and lesional skin (n = 3) was imaged with the confocal microscope. The study was conducted according to the principles embodied in the Declaration of Helsinki. All participants provided written informed consent before participating. The study was conducted in Vienna (Department of Dermatology, Medical University of Vienna, Austria) after approval of the local ethics committee of the Medical University of Vienna and the Austrian health authority (Bundesministerium für Gesundheit, Vienna, Austria; EK Nr: 801/2010; Eudra CT: 2010-022829-15).

### Spectroscopy of ICG

The stock solution of ICG (5 mg/ml) was diluted with distilled water to a final concentration of 3 µg/ml and the absorbance was measured with a Hitachi U-2000 spectrophotometer from 700 nm to 850 nm. The same solution was used for fluorescence emission scanning using a Hitachi F4500 fluorometer. Excitation was set to 633 nm (band width: 10 nm) due to the weak light emission of the lamp at higher wavelengths. Emission was recorded from 700–900 nm with a slit width of 20 nm, a PMT gain of 950 V, an integration time of 2 sec and a scan speed of 60 nm/min. Fluorescence and absorbance spectra were normalized to a peak intensity of 1.

### *In vivo* fluorescence/reflectance confocal microscopy

Fluorescence images were obtained using a commercially available confocal laser scanning microscope (VivaScope® 1500 Multilaser [Lucid Inc, Rochester, New York; USA]). A comprehensive review of the optical principles of the reflectance-only VivaScope® has been published [8], [23]. The multilaser system differs by the fact as it is equipped with three lasers with wavelengths of 488 nm (blue), 658 nm (red) and 785 nm (near-infrared), and three corresponding filter sets. In this study, we used the 488 nm laser (for fluorescein) in combination with a 550 nm emission filter (band width 88 nm) and the 785 nm laser (for ICG) combined with a 832 nm emission filter (bandwidth 40 nm). Each filter set is mounted in a bar, which can be set to three different positions: detection of all returning light, only reflected light, or only fluorescence emission. In all filter settings, a pinhole attenuates the light from out-of-focus planes. The scanned field of view is 500 µm×500 µm, producing images of 1000×1000 pixels. Axial resolution (section thickness) is <5 µm. Movement of the objective lens laterally with respect to the skin surface, enables to image at different horizontal directions (x and y axis) within the tissue; movement in the z-plane allows imaging at different depths of the tissue in a confocal mode due to the pinhole thereby generating an optical section. Depth measurements can be obtained from the Z-axis precision stepper motor, by indicating zero at the most superficial layer before scanning vertically into the tissue.

For making use of the fluorescence capabilities of the imaging system, we used either fluorescein or ICG as fluorescent dyes serving as contrast agents for labeling of human skin structures. 20 µl of a 0.5% solution of ICG (ICG-Pulsion, Pulsion Medical Systems, Munich, Germany) or a 0.08% solution of Sodium fluorescein (Thilorbin® eye drops, Alcon Pharma, Freiburg, Germany) were injected intradermally under standardized conditions using a 0.3 ml insulin syringe fitted with a 30-gauge needle. The syringe was placed at an angle of 5 to 10 degrees with respect to the skin surface. In case of fluorescein, imaging was performed before, immediately, 20, 40, 60, and 120 minutes after injection.

**Figure 1:**
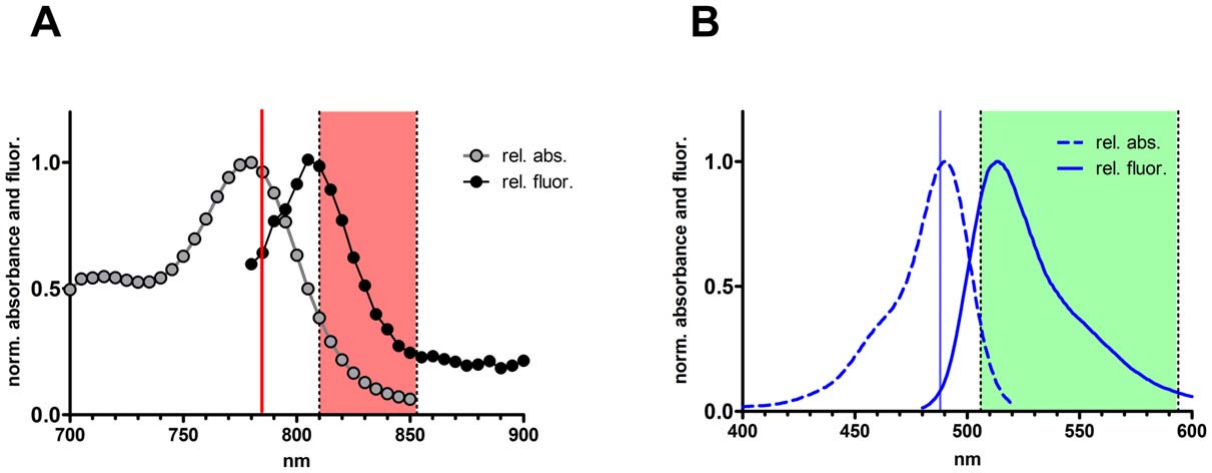
Absorbance and fluorescence emission spectra of ICG and fluorescein. A) ICG was dissolved in distilled water at a concentration of 3 µg/ml and the absorbance (grey cycles) was measured on a Hitachi U-2000 spectrophotometer. The same solution was used to record an emission wavelength scan of ICG on a Hitachi F4500 fluorometer (at an excitation of 633 nm). Spectra are normalized to the peak values. The laser line used for confocal fluorescence microscopy is indicated with a red line; the band of the detection channel is marked by a red transparent rectangle. B) Absorbance (dashed line) and fluorescence (continuous line) spectra of fluorescein. The exciting argon-laser line (488 nm) of the microscopy device is indicated by a blue line. The band of the detection channel is indicated by a green transparent rectangle. Data was derived from the Invitrogen spectra database (http://www.invitrogen.com/site.gateway.html?type=spectra&fileId=1300ph9).

Scanning after tissue administration of ICG was performed at the same time points and additionally 4, 8, 24, and 48 hours after its application. The ICG injection site serving as photobleaching control was light-protected with a bandage (Curapor®, 7×5 cm) for 48 hours. The standard procedure for *in vivo* scanning with the VivaScope® has been described previously [23]. Fluorescein has a maximum absorbance at a wavelength of 490 nm and the fluorescence emission is peaking at about 520 nm, which is ideally suited for the 488 nm laser. ICG has a maximum absorbance at a wavelength around 780 nm and its fluorescence emission peaks at about 805 nm (see Fig. 1), which is matching the 785 nm near-infrared laser excitation. Automatic image control was active, which allows the VivaScope® to automatically optimize the laser power so that the image is displayed with the proper illumination. Representative images at different layers of the epidermis and the dermis were acquired by varying the imaging depth from stratum corneum to a maximum depth of 200 µm in increments of 4.5 µm (Vivastack®). To study the kinetics of ICG imaging was performed in fluorescence mode at the defined time points for 9 participants. Fluorescein was additionally administered to five of those to evaluate the differing fluorescent properties of both components *in vivo*. Pathological skin morphology was imaged analogously in reflectance and fluorescence mode.

### Image analysis

Images recorded with the Vivascope® Multilaser system were imported to the free NIH image analysis package *ImageJ* as stacks. Representative regions of interest were defined within the rhombic fields (*Areolae cutaneae*) and the mean fluorescence intensities as well as standard deviations (reflecting the contrast) where measured for all the slices in the 3D stack using the measure stack plugin. Results were copied to MS-Excel™. The mean intensity of the deepest layer containing only background fluorescence was subtracted from the fluorescence intensities and values of 3–5 measurements were averaged. These values were determined for nine individuals receiving ICG and for five patients injected with fluorescein. For high quality graphs data were exported to GraphPad Prism-5.01™.

### *CellProfiler 2.0* Analysis

Automated image analysis, cell recognition and cell measurements were performed with the CellProfiler 2.0 open software [24]. First, all images were inverted so that the cell border (stained by ICG) was darker than the center of the cell, which was a prerequisite for automated cell recognition. After loading of images, objects were identified for a diameter between 10 and 60 pixels using the Otsu Global thresholding method with two-class weighting and a threshold correction factor of 0.7. Clumped objects were distinguished based on intensity. Identified objects (cells) were measured for size and shape parameters and values exported to a database, which was subsequently used for *CellProfiler-Analyst* [25] to generate a density plot for all the measured objects.

## Results and Discussion

Here, we studied the potential use of ICG for *in vivo* fluorescence microscopy using a novel multilaser equipment. There existed an initial lack of clarity regarding the peak absorbance of ICG, whereby previous publications claim this to be in the range of 780–810 nm depending on the solvent solution [26], [27]. This wavelength broadness prompted us to re-evaluate the absorbance and emission properties. The absorbance of ICG measured by spectrophotometry showed a peak at 780 nm, making the dye suitable for excitation by the 785 nm laser of the multilaser device. The emission curve was recorded with a spectrofluorometer and a peak was detected at 805 nm, which indicated that a substantial part of the fluorescence reaches the detector after passing the band-pass filter of the equipment (Fig. 1). These data qualified ICG for morphological fluorescence analysis in our present study. The fluorescence properties of ICG and its general approval for use in humans encouraged us to investigate its applicability as an *in vivo* fluorescence marker along with fluorescence confocal microscopy. To that end, we injected approximately 20 µl of the dye solution intradermally. At this low volume a potential rupture of the tissue structure is prevented.

**Figure 2:**
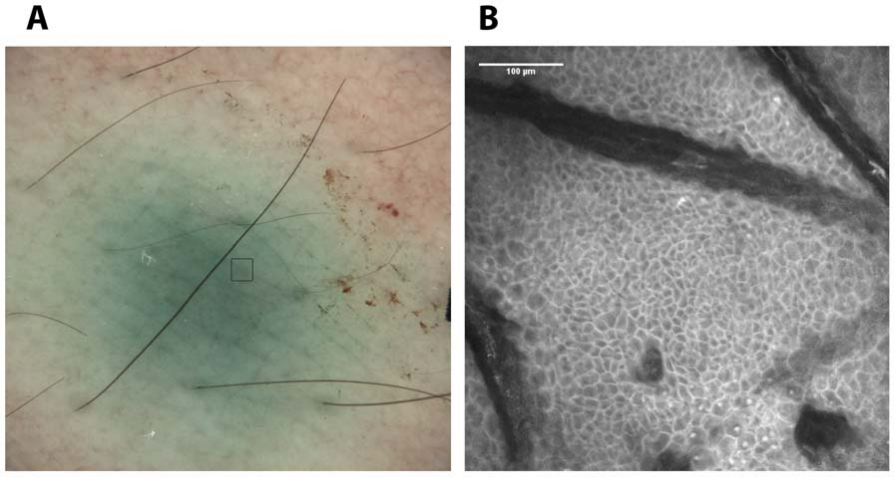
Macroscopic image of the ICG injection site and representative confocal fluorescence image. A) The injection site was imaged immediately after injection of 20 µl ICG solution by a dermatoscopic camera included in the Vivascope system. The square in the green injection area indicates the site of fluorescence microscopy shown in B. B) Representative confocal fluorescence image at a depth of 18 µm, 20 min after the injection.

Injection of ICG was reported as a short – time tolerable burning sensation at the injection site, resulting in green tincture of the skin with a visibility up to 96 hours (Fig. 2A). Fluorescein application resulted in a slightly lower pain sensation and a comparatively short-lived yellow coloration of the skin (data not shown). Confocal laser scanning microscopy was performed immediately after the injection and at different time points up to 48 h. In general, a distinct lucid intercellular fluorescence could be observed, which clearly demarcated the borders of the cells at the various depths of the optical sectioning thereby allowing for accurate assessment of the epidermal morphology (Fig. 2B).

**Figure 3:**
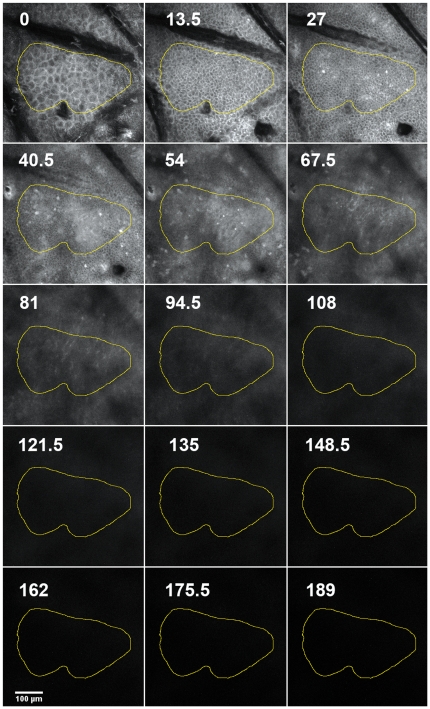
Montage of an image stack. Every third image of an image stack (site depicted in previous Fig. 2B) is shown with the depth of the slice (in µm) indicated by white numbers. The yellow boundary indicates a representative area of image analysis.

Since the device allows a vertical step size of 4.5 µm, image stacks were acquired that recorded epidermal and dermal layers down to 200 µm. These image stacks were used for quantifying fluorescence in representative, laterally homogenous regions (Fig. 3) and in addition, could also be used for calculating a 3D-projection of the skin (see Fig. S1).

**Figure 4:**
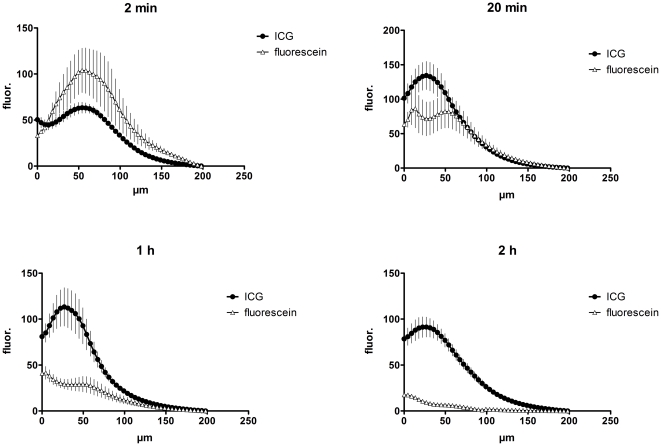
Quantification of the fluorescence intensities at different time points after fluorescein or ICG injection. Fluorescence intensities of representative areas (as shown in Fig. 3) were measured with ImageJ as described in the Methods section and are plotted against the depth of the image slice in µm. For fluorescein five individuals were imaged with three regions, each (n = 15), for ICG nine individuals were tested with three regions, each. Error bars represent SEM.

**Figure 5:**
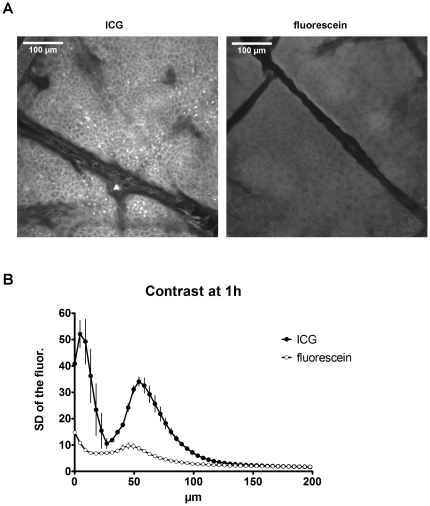
Comparison between fluorescein and ICG images with respect to contrast 1 h after injection. A) Representative images at 13.5 µm depth. B) Quantification of the contrast (as given by the SD of the fluorescence intensity).

**Figure 6:**
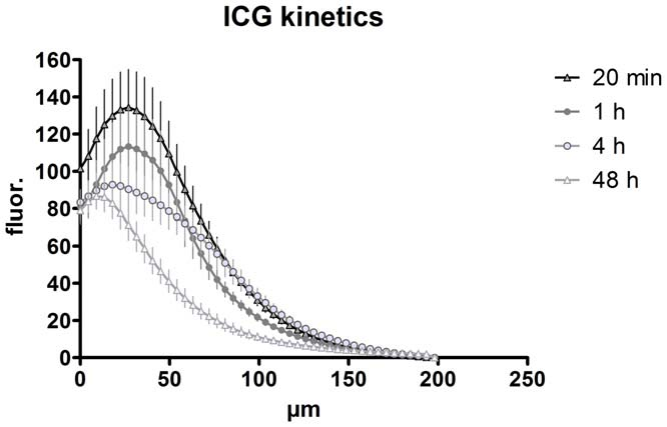
Kinetics of ICG fluorescence intensity up to 48 h. While fluorescein fluorescence was hardly detectable 2 h after injection, ICG fluorescence was still well visible 48 h after the injection. Four representative time points are indicated (n = 27, error bars represent SEM).

Assessing the time course of the fluorescence intensity in the different skin layers revealed a clear advantage of ICG over fluorescein. While the emission of fluorescein was higher than that of ICG briefly following injection, it declined quickly and was only faintly detectable after 2 h, whereas ICG fluorescence was still very prominent (Fig. 4). Immediately after the injection, the fluorescence maximum was confined to layers at the depth of the injection site around 60–80 µm beneath the skin's surface (Fig. 4, upper left panel). Reallocation of each dye to suprabasal layers was detected 20 min after application, when the initial signal of fluorescein already decreased. In contrast, the emission of ICG increased to the point that it exceeded the original fluorescein intensity (Fig. 4, upper right panel). One hour after application, ICG emission had slightly declined while that of fluorescein dropped to approximately 30–50% of its initial value (Fig. 4, lower left panel). Two hours after injection, fluorescein had already faded, while ICG fluorescence was still very distinct, showing just a minor decrease from the peak value at 20 min (Fig. 4, lower right panel). The advantage of ICG compared with fluorescein was not only evident for the total fluorescence intensity but also for the contrast that could be obtained in the various epidermal and dermal layers. One hour after injection, ICG fluorescence exhibited a prominent contrast, whereas the images obtained with fluorescein as tracer appeared dim and flat (Fig. 5A). A quantification of the contrast via the standard deviation of the fluorescence intensity revealed a much better performance of ICG as compared to fluorescein with a peak at 4.5 µm depth and a second one at 54 µm beneath the skin surface (Fig. 5B). The first peak appears to contain some light reflected by the stratum corneum. These data clearly show that ICG performs considerably better than fluorescein starting from the time point of 20 minutes after dye injection throughout the possible comparison period of 2 hours. Based on the striking stability and persistence of the ICG fluorescence as compared to that of fluorescein, we evaluated its signal up to 48 hours after injection. At that time the peak intensity had declined to approximately 65% of the maximum reached at 20 min (Fig. 6). However, the signal after 48 h still exhibited sufficient contrast and brightness for morphological assessment and was clearly superior to images obtained in reflectance mode. These data indicate that ICG is an efficient *in vivo* fluorescent dye in the skin, which allows flexible injection timing prior to morphological evaluation via fluorescence microscopy in a routine clinical setting.

**Figure 7:**
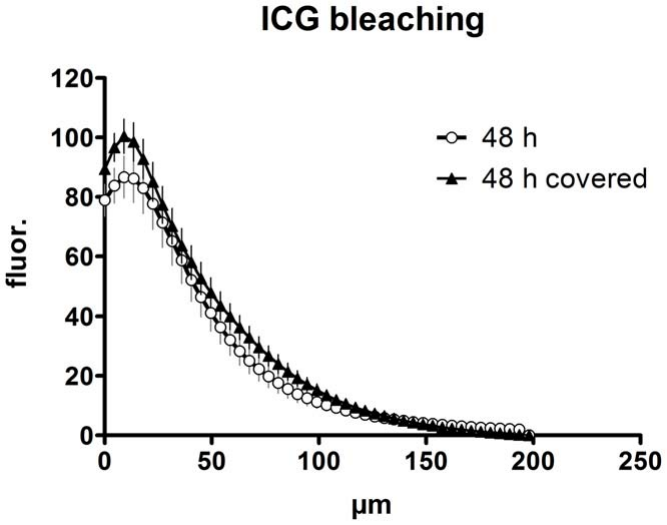
There is only a limited extent of bleaching within 48 h. ICG fluorescence is shown 48 h after injection for an area that was exposed to daylight and subject to repetitive scanning (at 2 min, 20 min, 1 h, 2 h, 4 h, 8 h, 24 h and 48 h) – or an area that was covered for 48 h before imaging to protect it from bleaching.

Moreover, we investigated whether ICG is subject to photobleaching – a common problem associated with fluorescein. To that end we investigated uncovered ICG-contrasted skin sites that were scanned at different time points up to 48 h (Fig. 7; legend) and compared them to a control ICG-injected site that was covered during the whole time period with a non-transparent bandage. The effect of photobleaching was negligible, resulting in just a minor reduction to approximately 87% of the control value (Fig. 7). This indicated that repetitive scans after single ICG injections have almost no impact on the fluorescence signal, allowing for repeated morphological assessment via FCM of the same skin site, for instance to monitor therapy efficacy.

**Figure 8:**
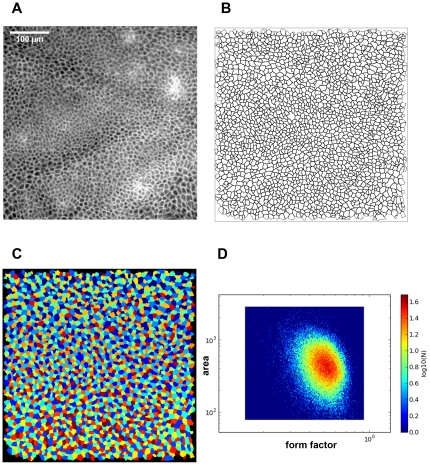
Automated cell recognition and analysis using *CellProfiler*. A) Representative confocal fluorescence image of the stratum spinosum after ICG-injection. B) Outline of the cell borders after the cell recognition by the *CellProfiler* software. C) Objects identified by *CellProfiler*. D) Density plot of cell area versus form factor (circularity) after analysis of 108000 objects (from 59 images) using *CellProfiler Analyst*.

The well-defined cellular structure that could be observed after ICG injection by confocal fluorescence microscopy *in vivo* encouraged us to test whether the cellular staining pattern could be exploited for automated quantitative high-content imaging. This is a method that extends the potential of microscopy to tissue cytometry, by comprising analysis of thousands of cells, which allows statistics or gating of cell populations similar to flow cytometry [28]. We achieved this using the free software *CellProfiler* [24], [29] that allows for automatic loading of images and object identification followed by quantitative analysis. Fluorescence images with ICG staining (Fig. 8A) had to be inverted for proper cell recognition. Using the parameters described in the Methods section, adjacent cells were correctly separated (Fig. 8B), automatically recognized as objects (Fig. 8C), and measured for size and shape parameters. *CellProfiler* allows saving of the data for thousands of objects and exporting of the values as a database, which can be loaded in a companion software, the *CellProfiler Analyst* [25]. This software then provides professional data visualization and statistics tools – such as density plots to illustrate correlations between different parameters (Fig. 8D).

**Figure 9:**
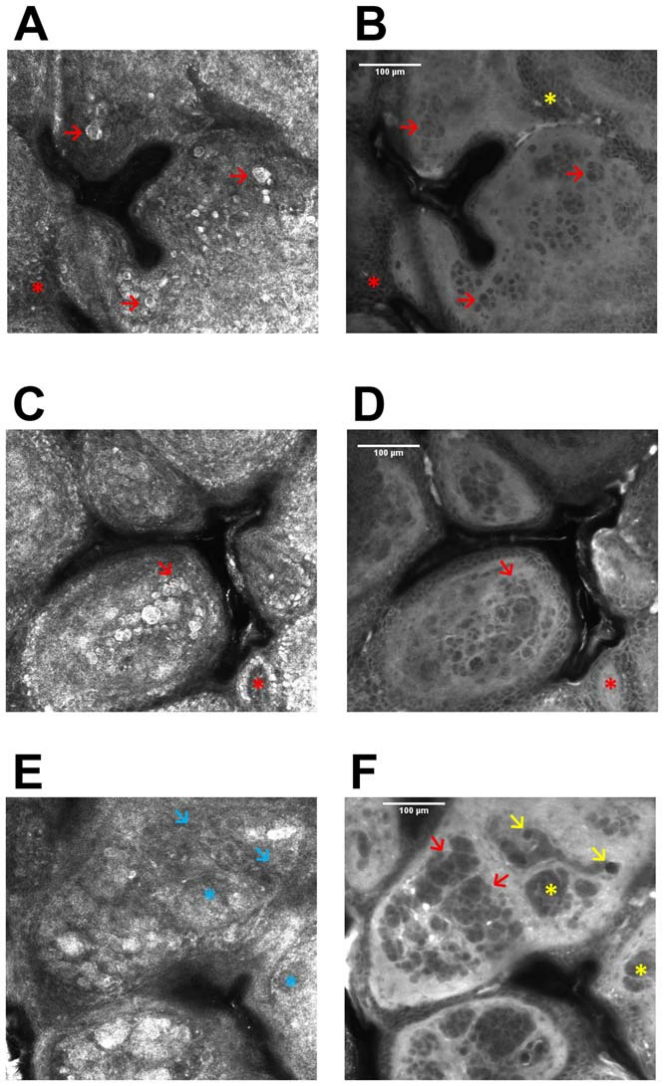
*In vivo* confocal laser scan of a dermal nevus after intradermal ICG injection. A) and B) Scan of a dermal nevus 33 µm below the stratum corneum in reflectance mode (A) and in fluorescence mode (B): red arrows are indicating uniform bright cells with bright cytoplasm and central small dark nuclei (A); fluorescence mode reveals a negative pattern of these nevocytes (B); red asterisks are indicating keratinocytes of the stratum spinosum in both modes (A and B); in fluorescence mode (B) the intercellular distribution of the dye exhibits a well-defined morphology of singular cells in the stratum spinosum which can only be evaluated in the fluorescence mode; yellow asterisk is indicating a precise cellular pattern which cannot be detected in the reflectance mode scanning. C) and D) Confocal scan of a dermal nevus 34.5 µm below the stratum corneum in reflectance mode (C) and in fluorescence mode (D); dermal cell clusters are indicated by arrows; (C) displays bright cells and (D) a negative pattern of these cells; asterisks are indicating a dermal papilla: revealing a hyperrefractile dermal papillary ring (C) but this edged papilla [37] cannot be detected in fluorescence mode (D). E) and F): Laser scan of a dermal nevus 63 µm below the stratum corneum in reflectance mode (E) and in fluorescence mode (F); dermal cell clusters are indicated by red arrows and yellow asterisks revealing a sharp negative pattern of nevocytes (F); yellow arrows are indicating blood vessels (F) ; blue arrows and blue asterisks are indicating the corresponding sites (E) in comparison to the yellow symbols (F) displaying no detectable signal of cell structures.

**Figure 10:**
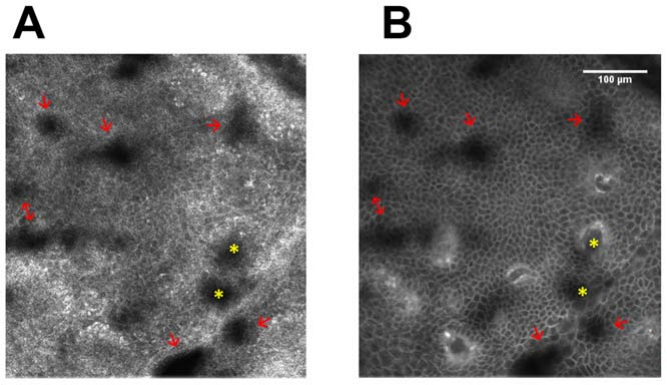
*In vivo* confocal laser scan of a contact dermatitis area. A) and B): Laser scan of an irritant contact dermatitis at the spinous layer/DEJ in reflectance mode (A) and in fluorescence mode (B) after ICG injection intradermally; epidermal spongiosis is indicated by red arrows (A and B); a yellow asterisk marks a structure, which is indistinguishable from spongiosis in the reflectance mode (A) but which is clearly identified as dermal papilla in ICG-mediated fluorescence microscopy (B).

After having demonstrated that ICG is well suited as contrast agent in fluorescence microscopy of the skin *in vivo*, by providing a resolution down to the sub-cellular level, we were interested in testing its applicability for pathological assessment of lesional skin. For that purpose we investigated samples of intradermal nevi, irritant contact dermatitis and KTP-laser treatment induced necrosis. This was performed 90 min after injection of ICG using both reflectance and fluorescence mode microscopy. The reflectance mode image of the dermal nevus 33 µm below the skin surface demonstrated uniform lucid cells with bright cytoplasm and central small dark round nuclei (Fig. 9A) as already described before [30], [31], [32]. Due to the mere intercellular localization of the dye, the nevoid cells appear in a negative staining pattern (Fig. 9B), which allows for a precise morphological analysis. Furthermore, the intercellular distribution of ICG uncovers the morphology of single keratinocytes of the stratum spinosum (Fig. 9A). In reflectance mode scanning, it is not possible to reach a comparable resolution of these cellular details. Dermal nevus cell nests are also depicted in a negative pattern (Fig. 9D). A dermal (edged) papilla with a hyperrefractile dermal papillary ring composed of melanocytes and melanin-rich keratinocytes is illustrated in reflectance mode (Fig. 9E, asterisk). These cells cannot be revealed by means of fluorescence because backscattered light from refractive structures in reflectance mode is blocked by the fluorescence band-pass filter. Fluorescence microscopy exhibits the dermal papilla as a lightish round topology interposed among deeper epidermal layers displayed in an intercellularly staining pattern (Fig. 9D). Fig. 9E is clearly inferior in comparison to Fig. 9F as dermal clusters of nevocytes become entirely apparent in fluorescence images, and blood vessels are detectable only in this setting. In case of the dermal nevus fluorescence scanning provides additional morphological information in form of accurate resolution of dermal cell clusters throughout the entire lesion. The possibility to picture dermal vessels in the fluorescence mode offers the assessment of vascular changes in a variety of skin pathologies as well as along with treatment. The linkage of reflectance and fluorescence mode permits the combination of the advantages of each technique. Evaluation of melanin-rich keratinocytes solely via reflectance mode and estimation of dermal vessels exclusively via fluorescence mode exemplify synergistic morphological information. Another pathological condition that we investigated by ICG-based *in vivo* fluorescence microscopy is irritant contact dermatitis leading to eczema formation. Disruption of keratinocytes (spongiosis) is one of the key features of eczematous skin, which is represented in Fig. 10A and B [33], [34], [35]. In both modes several sites of spongiotic areas are visible revealing dark, round to oval structures in reflectance mode (Fig. 10A) and a gradual disappearance of the cell borders visible in fluorescence mode due to the extracellular dye (Fig. 10B). The fluorescence mode provides a clear advantage over reflectance mode, as dermal papilla often appear equally as roundish dark areas in reflectance mode similar to spongiotic areas, whereas they are clearly distinguishable in fluorescence mode elucidating the morpholopical disparities by *in vivo* microscopy without the need for histological analysis of biopsies.

The dermal papilla displayed in the reflectance mode (Fig. 10A) corresponds to a “black papilla” [36]. In fluorescence mode these papillae at the dermal/epidermal junction (DEJ, Fig. 10B) are displayed as round, dark structures surrounded by a lucid rim caused by circumferentially arranged basal cells and solely dyeing of the extracellular space. The DEJ first appears 55–65 µm below the corneal layer, depending on the body site [37]. Fig. 9D shows another case of a dermal papilla at a scanning depth of 34.5 µm below the stratum corneum.

**Figure 11:**
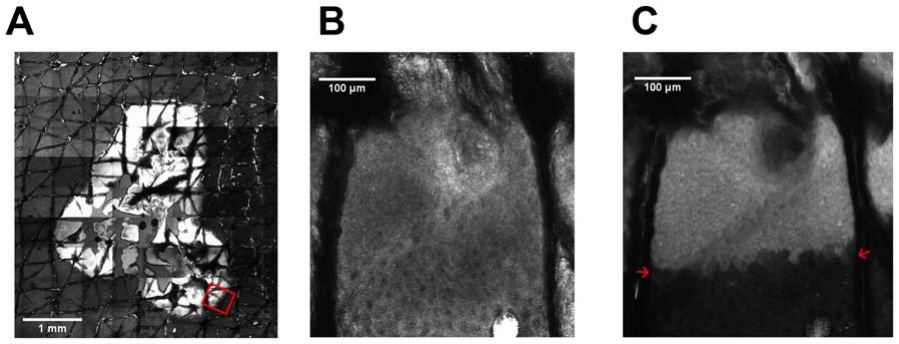
Confocal microscopy of a necrotic lesion. A): Mosaic image of stitched scans of a necrosis due to KTP-laser treatment 7.5 µm below the stratum corneum after ICG injection intradermally in fluorescence mode; the red square indicates the scanning location for (B) and (C); B) reveals a normal honeycomb pattern of the stratum granulosum in reflectance mode; C) the border of necrotic and viable cells is clearly visible by the strong fluorescence of the necrotic cells, which lost their barrier function (the border is indicated by red arrows in the fluorescence mode.

Furthermore, we assessed an example of a necrotic skin area by ICG-based *in vivo* microscopy. A fluorescence mosaic scan of a necrotic lesion induced by KTP-laser treatment is shown 7.4 µm below the skin surface (Fig. 11A). The necrotic area is delimited more precisely in fluorescence as compared to reflectance mode (data not shown). Fig. 11B and C present a higher magnification of the lesion, which clearly demonstrates the advantage of the fluorescence microscopy. In reflectance mode, a normal honeycomb pattern indicating the undamaged keratinocytes can be revealed, whereas necrotic cells appear rather cloudy. However, the difference is not easily visible in this mode (Fig. 11B). In contrast to that, the fluorescence mode (Fig. 11C) displays a very clear border between necrotic and viable cells as the dye is taken up by necrotic cells due to the loss of barrier function.

Altogether our data clearly show that the combination of ICG as fluorescent tracer with confocal *in vivo* fluorescence microscopy offers a number of significant advantages for dermatological diagnoses. First, it provides a minimal invasive method for morphological assessment of healthy or diseased skin through bypassing the need for biopsies and histology. It is evident that this is also expanding the possibilities to investigate the same skin samples at different time points so as to evaluate the efficacy of treatment or the progression of a skin disease. Second, it is clearly superior to fluorescein as fluorescent marker, based on much longer fluorescence emission *in vivo* and higher photostability. Third, the distinct cellular staining pattern that is possible with ICG-mediated fluorescence as compared to the routinely used reflectance microscopy allows the application of sophisticated automated cell recognition and measurement approaches extending the microscopy investigation from a mere qualitative method to a quantitative measurement tool. Finally, our results provide evidence that this method of skin imaging allows a clear diagnostic evaluation of pathologies exceeding currently used reflectance microscopy.

## Supporting Information

Figure S1 **3D projection of an ICG-fluorescence stack.** Images of an ICG-injected area were recorded with an increment of 4.5 µm from the stratum corneum to a depth of 200 µm. These images were loaded to the *ImageJ* software as a stack, followed by calculation of a 3D-projection using the “3D project” function of ImageJ,
(GIF)
