# Supplementary figures and images for: Intradermal Indocyanine Green for In Vivo Fluorescence Laser Scanning Microscopy of Human Skin: A Pilot Study
Source: PLoS One. 2011 Aug 31;6(8):e23972. doi: 10.1371/journal.pone.0023972 (PMC3164142; doi:10.1371/journal.pone.0023972)

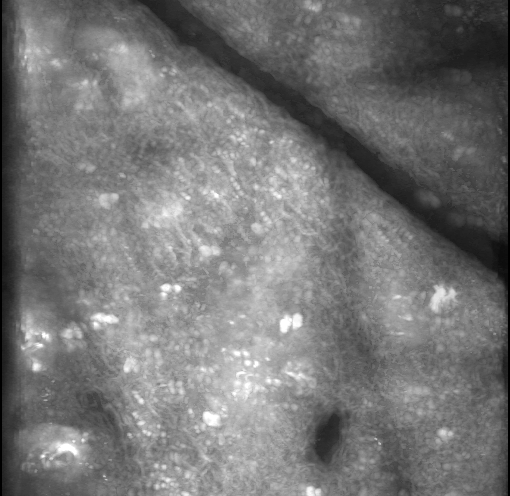

Supplement: Figure S1 — 3D projection of an ICG-fluorescence stack. Images of an ICG-injected area were recorded with an increment of 4.5 µm from the stratum corneum to a depth of 200 µm. These images were loaded to the ImageJ software as a stack, followed by calculation of a 3D-projection using the “3D project” function of ImageJ, (GIF) [file pone.0023972.s001.gif]
